# Topological Data Analysis as a New Tool for EEG Processing

**DOI:** 10.3389/fnins.2021.761703

**Published:** 2021-11-03

**Authors:** Xiaoqi Xu, Nicolas Drougard, Raphaëlle N. Roy

**Affiliations:** ^1^ISAE-SUPAERO, Université de Toulouse, Toulouse, France; ^2^ANITI–Artificial and Natural Intelligence Toulouse Institute, Université de Toulouse, Toulouse, France

**Keywords:** topological data analysis (TDA), electroencephalography (EEG), persistent homology, brain-computer interface (BCI), machine learning

## Abstract

Electroencephalography (EEG) is a widely used cerebral activity measuring device for both clinical and everyday life applications. In addition to denoising and potential classification, a crucial step in EEG processing is to extract relevant features. Topological data analysis (TDA) as an emerging tool enables to analyse and understand data from a different angle than traditionally used methods. As a higher dimensional analogy of graph analysis, TDA can model rich interactions beyond pairwise relations. It also distinguishes different dynamics of EEG time series. TDA remains largely unknown to the EEG processing community while it fits well the heterogeneous nature of EEG signals. This short review aims to give a quick introduction to TDA and how it can be applied to EEG analysis in various applications including brain-computer interfaces (BCIs). After introducing the objective of the article, the main concepts and ideas of TDA are explained. Next, how to implement it for EEG processing is detailed, and lastly the article discusses the benefits and limitations of the method.

## 1. Introduction

Electroencephalography (EEG) records brain electrical activity in a non-invasive way and contains rich information about the underlying brain state and function. It is intensively used in diagnosis and analysis of various neurological disorders such as epilepsy, schizophrenia and autism spectrum disorder (ASD) (van der Stelt and Belger, 2007; Billeci et al., 2013; Acharya et al., 2015) as well as for non-clinical applications such as sport and sleep monitoring (Borbély et al., 1981; Thompson et al., 2008).

A key step in EEG processing is to extract relevant features or markers for the considered application. Many techniques have been developed, ranging from traditional spectral analysis, to non-linear analysis, as well as to recent deep learning techniques (Muthuswamy and Thakor, 1998; Müller et al., 2008; Murugappan et al., 2008; Subha et al., 2010; Craik et al., 2019). Since the brain is a huge network of neurons wired together and its function is based on the synchronization of neurons, it is natural to study EEG signals using functional connectivity metrics as features (Sporns, 2013). Most of the current work use graph theory as tools to extract features e.g., small-worldness, global clustering coefficient and characteristic path length (Ismail and Karwowski, 2020). While being powerful tools, graph models oversimplify the interactions between neurons by reducing them to nodes and edges, thus capturing only low-dimensional information (0 and 1). In contrast, topological data analysis (TDA) allows to explore higher-dimensional information by using higher dimensional representations called simplicial complexes (a set of points, segments, triangles and their higher dimensional analogs, cf. section 2.1 for a formal definition).

Although it is not novel to classify time series by extracting topological features using TDA (Seversky et al., 2016; Umeda, 2017), it is only recently that this technique has attracted attention in the domain of EEG processing, especially for clinical applications. Some pioneering work has shown that topological features extracted from EEG signals reveal relevant information for various neurological disorders (Wang et al., 2018; Ibáñez-Marcelo et al., 2019; Yamanashi et al., 2021).

In this mini review, we aim to identify where and how TDA can be used for EEG processing by reviewing the current literature, and point out potential directions for future work. The organization is as follows: section 2 is a short presentation of the main principle of TDA; section 3 summarizes various ways to apply TDA in EEG processing based on the current literature; section 4 discusses the advantages and limitations of TDA for EEG analysis and the gaps in current research.

## 2. Topological Data Analysis

Topological data analysis is a young but rapidly growing domain at the intersection of algebraic topology and data science. There already exist some good tutorials for non-mathematicians like data scientists or neuroscientists (Sizemore et al., 2019; Chazal and Michel, 2021). For a more mathematical introduction to algebraic topology, the books of Hatcher (2000) and Ghrist (2014) could be a good starting point. In this section we briefly describe the main idea of TDA and refer the interested readers to the above references. All the definitions are gathered in section 2.1 to facilitate the reading.

### 2.1. Definitions

**DEFINITION 1.** *Two functions f, g* : *X → Y are said to be **homotopic** if there exist a continuous function H* : *X* × [0, 1] → *Y such that H*(·, 0) = *f and H*(·, 1) = *g*. *Two topological spaces X and Y are* ***homotopic*** *if there exist continuous functions f* : *X* → *Y and g* : *Y* → *X such that f* ◦ *g and g* ◦ *f are homotopic to id_Y_ and id_X_, respectively*.

**DEFINITION 2.** *A **k-simplex**, noted as* Δ_*k*_ = [*v*_0_, …, *v*_*k*_], *is the convex hull of a set of k* + 1 *linearly independent points*.

**DEFINITION 3.** *A **simplicial complex** is a collection of simplices satisfying following conditions: every subset and their intersections are also simplices in the collection*.

**DEFINITION 4.** *The **boundary** of a *k*-simplex* Δ_*k*_ = [*v*_0_, …, *v*_*k*_] *is defined as an alternating formal sum of* (*k*−1)-*simplices*, $\partial_{k}\Delta_{k}=\underset{i}{\sum }{\left(-1\right)}^{i}\left[v_{0},\ldots ,\hat{v_{i}},\ldots ,v_{k}\right]$ *where* $\hat{v_{i}}$ *means omitting v_i_*.

**EXAMPLE 1.** *Take a 2-simplex* Δ_2_ = [*v*_0_, *v*_1_, *v*_2_] *for example*: ∂_2_Δ_2_ = [*v*_1_, *v*_2_] − [*v*_0_, *v*_2_] + [*v*_0_, *v*_1_] = [*v*_1_, *v*_2_] + [*v*_2_, *v*_0_]+[*v*_0_, *v*_1_]. *Its boundary is in fact the loop formed by its edges*.

**REMARK 1.** *A simple yet important property is that the boundary of a boundary is always zero*: ∂_*k*_ ◦ ∂_*k*+1_ = 0, ∀*k* ≥ 0. *In other words*, im ∂_*k*+1_ ⊆ ker ∂_*k*_. *Homology is defined based on this property*.

**EXAMPLE 2.** *To have an intuition of what goes on here, we could take the same example above*. ∂_1_ ◦ ∂_2_ Δ_2_ = ∂_1_([*v*_1_, *v*_2_] − [*v*_0_, *v*_2_] + [*v*_0_, *v*_1_]) = *v*_2_ − *v*_1_ − *v*_2_ + *v*_0_ + *v*_1_ − *v*_0_ = 0.

** DEFINITION 5**. *The k-dimensional **homology** of a simplicial complex C is defined as the quotient space H_k_*(*C*): = ker ∂_*k*_/ im ∂_*k*+1_, *in which two elements that differ by a k-boundary are considered as the same element. The dimension of H_k_*(*C*) *is called the k^th^ **Betti number***.

### 2.2. Topology and Homology in a Nutshell

Topology is the mathematical branch which studies the properties that are preserved by continuous deformation, such as scaling, twisting but not tearing. It could be viewed as “geometry without metric.” Distance is of no importance in topology, instead the whole theory is based on the notion of “closeness.” More precisely, what we call topological properties are those invariant under homeomorphism (continuous map whose inverse is also continuous). However, homeomorphism is often too strict. A looser but useful notion is homotopy (see Definition 1). It is not only useful in the theory, but also in practice since a lot of noise in the real world data can also be viewed in a homotopic way, such as an blurred edge is homotopic (but not homeomorphic) to the ideal edge in a digital image.

Based on the notion of homotopy, homotopy groups can be defined and distinguish different topological spaces. But they are difficult to compute in general. An alternative notion is homology (see Definition 5), which is based on simplicial complex (see Definition 3) and can be computed effectively using the “divide and conquer” strategy.

Intuitively, the *k*-dimensional homology catches *k*-dimensional “holes,” i.e., independent cycles that are not filled. Homology is a homotopy invariant and the dimensions of the homology group, i.e., Betti numbers, are among the first topological statistics applied in real world applications (Giusti et al., 2015).

### 2.3. Persistent Homology

What makes TDA powerful and particularly suitable to capture hierarchical features is persistent homology, a method that extracts persistent topological features across scales. The key notion is filtration, which is a nested family of subcomplexes indexed by a parameter. The homology of these subcomplexes evolves as the parameter grows, giving rise to the barcode or persistence diagram as a description of persistence of connected components (described with components' birth and death) by 0-dimensional homology, and of multidimensional holes by higher dimensional homology.

**Figure 1:**
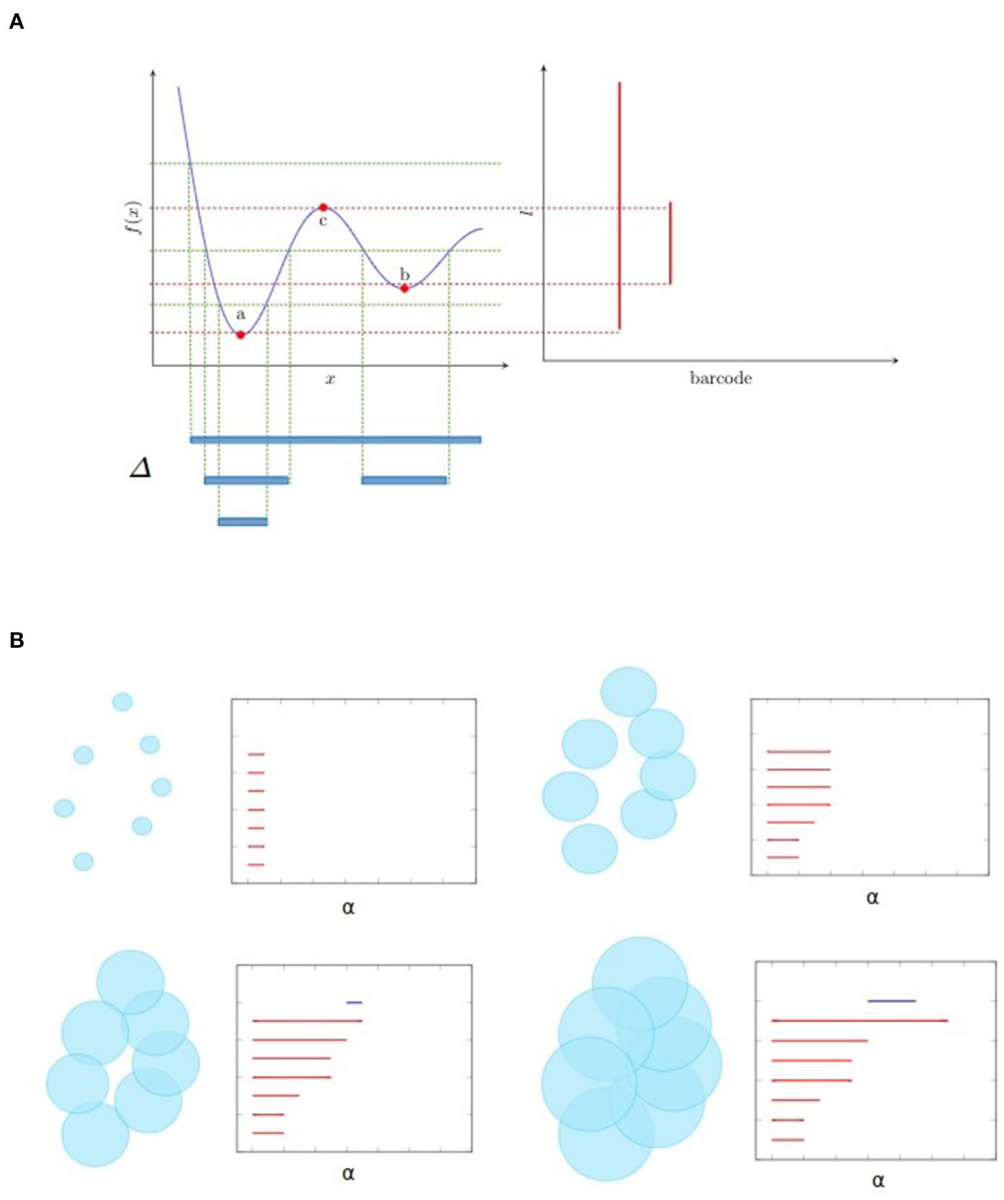
Examples of filtration and the associated barcode. **(A)** shows the sublevel filtration of function *f*(*x*). The first and second connected component appear at point *a* and *b* respectively and merge together at point *c*, giving rise to two bars: one from *a* till infinity and the other from *b* to *c*. **(B)** shows several stages of Rips filtration. At first, all points are isolated and we have the same number of bars and points. As balls grow larger, some balls start to merge together, giving death to certain bars. Then one loop appears, marked as a 1-dimensional feature corresponding to the blue line in the barcode. Finally the loop is filled so the blue bar stops and every point merges into one component living forever.

Figure 1 illustrates two kinds of filtration that are frequently used. Let *f* be a map from ℝ to ℝ as shown in Figure 1A. Then the *sublevel filtration* consists of the family of subcomplexes *S*_*l*_ = {Δ ∈ ℝ:*f*(Δ) < *l*}. For this example the only non trivial topological information is carried by a 0-dimensional homology since we have only segments (as shown at the bottom of Figure 1A) and no loops. We see as the parameter *l* increases, there are new components emerge, marked as birth, and also some component merges with the other one, leading to its death (by convention the elder rule is applied that keeps the older one). The parameters corresponding to birth and death form a family of intervals. Chazal et al. (2012) demonstrated that this family of intervals is unique up to reordering and can be used as a topological feature named *barcode*. We could also mark on the plan ℝ^2^ the point at which the *x* and *y* coordinates correspond to birth and death of a feature, respectively. These points, together with the diagonal, are called *persistence diagram*.

Figure 1B is an example of Rips filtration. Given a set of points *X* and a positive number α, the *Rips complex* Rips_α_(*X*) is the simplicial complex including all simplices in which the distance between any two of their vertices is smaller than α. *Rips filtration* consists of the family of Rips complex {_Rips_α_(*X*)}α_ indexed by α. In the example in ℝ^2^, we could imagine balls with increasing radius around the initial point cloud. As the balls become bigger, they merge with each other, leading to the death of certain connected components but also the birth of some loops (only one for the example in Figure 1B). The larger the scale of the loop is, the more persistent it is in the barcode. In most cases, long bars correspond to significant features while short bars correspond to noise. As α goes from 0 to ∞, the topology of each Rips complex goes from one trivial case, disjoint unions of points, to another trivial case, all points merged to one connected component. The persistence homology records the evolution between the two extreme cases, in the middle of which interesting features come to light. So, unlike hard thresholding methods usually used in the construction of graphs, TDA preserves more information.

## 3. TDA Applied on EEG Data

In order to retrieve and evaluate in a comprehensive manner all research works related to TDA and EEG processing, a systematic search was conducted as described here after. Hence, using (topological data analysis, TDA) and (electroencephalography, EEG) as search terms, we found 70 publications on PubMed and 85 publications on Web of Science. Then we examined the title and abstract of each non-redundant publication and excluded those concerning recording methods other than EEG (e.g., MEG, fMRI) or using graph theory analysis instead of TDA as tools. The remaining publications are summarized in Table 1.

**Table 1:** Summary of applications of TDA to EEG analysis (ordered by publication date).

**References**	**Domain**	**Transformation**	**Method**	**Features**	**Classifier**	**Dataset**
Altındiş et al. (2021)	MI-BCI	-	Time delay embedding	Persistence diagram	-	Graz dataset
Bischof and Bunch (2021)	Eyes-open/eyes-closed classification	-	Time delay embedding	Betti-numbers	CNN	Bonn dataset
Yamanashi et al. (2021)	Delirium	-	Time delay embedding	Area of the 1-dimensional Betti curve	-	Private dataset
Majumder et al. (2020)	Autism Spectrum Disorder (ASD)	-	Sublevel filtration	Persistent entropy	SVM	Private dataset
Wang et al. (2020b)	Aphasia	ICA	Sublevel filtration	Persistence landscape	-	Private dataset
Wang et al. (2020a)	Aphasia	ICA	Gradient filtration	Persistence landscape	-	Private dataset
Ibáñez-Marcelo et al. (2019)	Hypnotizability	ICA	Connectivity

We note that besides standard preprocessing steps, e.g., band-pass filtering, downsampling, and artifacts removal, few authors have chosen to transform data into another space. Independent component analysis (ICA) is the one mostly used with the purpose of getting source components, then followed by Fourier transform for the purpose of denoising data.

From the papers that were collected, three methods of employing TDA emerge: the first one applies it directly onto the EEG signals; the second one applies it onto the connectivity network; and the third one onto the phase space. For the first group, sublevel filtration illustrated in Figure 1A is applied directly on the EEG time series of each channel which is as the function *f* in the example. For the second group, Rips filtration, as illustrated in Figure 1B, is applied on the point cloud in which each point represents a channel or a source component, and the distance is measured by connectivity measures such as Pearson correlation. For the third group, even though it is possible to embed data spatially, all authors have followed time-delay embedding well backed by Takens' theorem (Takens, 1981) which gives the minimum dimension of embedding in order to reconstruct attractors (a set of points in the phase space that is invariant under dynamics and “attracts” neighboring points) up to diffeomorphism. Time-delay embedding of a time series {*x*(*t*)}_*t*_ is formed by keeping *k* observations before current time (*x*(*t*− *k* + 1), *x*(*t* −*k* + 2), …, *x*(*t* − 1), *x*(*t*)). In the phase space each point represents the state at a certain time. Takens' theorem guarantees that the differential, so topological, properties of the attractors are preserved by time delay embedding, which permits to distinguish different EEG time series based on the topology of the attractors in the phase space.

A barcode or persistence diagram is constructed along with the filtration process as shown in section 2.3. A metric structure is still needed in order to measure differences between barcodes or persistence diagrams. The Bottleneck distance, or Wasserstein distance, measures the distance between persistence diagrams by pairing points in the two diagrams.

There are many other ways to extract topological features from persistence diagrams other than measuring distance in the original space. The simplest way might be extracting Betti numbers. Another simple method is persistence entropy (Chintakunta et al., 2015) which gives a scalar description of the barcode. Additionally, persistence landscapes introduced by Bubenik (2015) smartly embed persistence diagrams into a Hilbert space where most machine learning algorithms can be applied, which makes them a popular choice.

Finally, after the feature extraction step, a classifier might be applied or not depending on the application at hand (e.g., diagnostic, active brain-computer interface, or mental state estimation via a passive brain-computer interface). The most used ones are linear classifiers due to the small size of clinical datasets. However, when the amount of data allows, deep learning methods show promising performance (Bischof and Bunch, 2021). TDA can also be combined with other frameworks such as Bayesian networks (Nasrin et al., 2019).

## 4. Discussion

TDA has many advantages. Firstly, topological features are by nature robust and invariant to transformations such as translation, amplitude and frequency scaling (Wang et al., 2018). Secondly, TDA is well suited for neuroscience, especially analyses involving connectivity networks. Neurons far apart can communicate with each other since some axons extend up to one meter or more, so it's how they are connected, i.e., the topological structure of the network, and not the distance that counts. Thirdly, TDA can capture global and higher dimensional features where other methods such as graph theory fails.

The main limitation of TDA stems also from its strength. Since it neglects all metric related information, this harms its ability to distinguish data of different categories. Considering the pros and cons of TDA, it is recommended to combine TDA with other methods to use it to its full potential. One promising direction is the combination of TDA with deep learning techniques. There have been some pioneering work e.g., the work of Carriere et al. (2020), Kim et al. (2020), and Royer et al. (2021). Much more still remains to be explored, especially leveraging the particular structure of EEG signals. Another direction is to associate TDA with statistics. The topological features could be seen not as deterministic but random variables. The notions such as convergence rate, consistency, confidence region etc. of the extracted topological features could be studied.

Although there are theoretical results showing the robustness of persistence diagrams under perturbations (Cohen-Steiner et al., 2007; Bubenik, 2015), in practice the technical details of applying TDA for EEG signals need to be further investigated. Altındiş et al. (2021) started in this direction by trying to find the optimal embedding dimension, time delay and time window size using false nearest neighbor (FNN) test. The paired *t*-test showed that the significance level of extracted topological features was very sensitive to the choice of embedding parameters and hence it was important to use the optimal parameters.

The current domain of application is still quite restricted to clinical studies to improve the diagnostic of neurological diseases. However, TDA is also quite suitable for other non-clinical EEG related areas, e.g., EEG based brain-computer interface (BCI; Wolpaw et al., 2000) which allows the explicit control of machines or implicit mental state estimation using only EEG signals. Further, the datasets used in current publications are mostly private datasets, which if possible should be replaced by publicly available ones for increased reproducibility and comparison with other work.

TDA is going through rapid development, both in theory and in application. With better theoretical foundation and more open source software and code published online^1^, we believe that it will become part of the arsenal of tools for a broader scientific community. Hopefully we will see more publications on EEG processing using TDA in the future.

## Author Contributions

XX: original idea and drafting of the article. ND and RR: writing supervision and critical revisions. All authors contributed to the article and approved the submitted version.

## Funding

The work was funded by ANITI (Artificial and Natural Intelligence Toulouse Institute), Toulouse, France.

## Conflict of Interest

The authors declare that the research was conducted in the absence of any commercial or financial relationships that could be construed as a potential conflict of interest.

## Publisher's Note

All claims expressed in this article are solely those of the authors and do not necessarily represent those of their affiliated organizations, or those of the publisher, the editors and the reviewers. Any product that may be evaluated in this article, or claim that may be made by its manufacturer, is not guaranteed or endorsed by the publisher.

## Footnotes

^1^For example see https://github.com/FatemehTarashi/awesome-tda for a list of various softwares and resources.
